# Interaction of low-density neutrophils with other immune cells in the mechanism of inflammation

**DOI:** 10.1186/s10020-025-01187-5

**Published:** 2025-04-09

**Authors:** Yu Fu, Zongmei Wen, Jie Fan

**Affiliations:** 1 Department of Surgery, School of Medicine, University of Pittsburgh, Pittsburgh, 15213 USA; 2 Department of Anesthesiology, Shanghai Pulmonary Hospital, School of Medicine, Tongji University, Shanghai, 200433 China; 3 Research and Development, Veterans Affairs Pittsburgh Healthcare System, Pittsburgh, PA 15240 USA; 4 Department of Immunology, School of Medicine, University of Pittsburgh, Pittsburgh, 15213 USA; 5 McGowan Institute for Regenerative Medicine, University of Pittsburgh, Pittsburgh, PA 15219 USA

**Keywords:** Low-density neutrophils, Inflammation, Macrophages, Platelets, T cells, B cells

## Abstract

Low-density neutrophils (LDNs) are a unique subpopulation of neutrophils, play a significant role in regulating innate and adaptive immunity in various inflammation-related diseases. Emerging evidence suggests that LDNs play a significant role in the pathogenesis and progression of various diseases, including infections, autoimmune disorders, and cancer. In this review, we address the origin, development, and heterogeneity of LDNs, and the roles of LDNs in system homeostasis and diseases. We will focus on the findings of the interaction between LDNs and other immune cells. We will then discuss potential novel therapeutic strategies of intervention in diseases by targeting LDNs.

## Introduction

Neutrophils, originating from the bone marrow, are the most abundant immune cells in human circulation (Fliedner et al. 1964; Burn et al. 2021). Emerging studies have revealed neutrophil phenotypic and functional heterogeneity (Ng et al. 2019; Hedrick and Malanchi 2022). Of note, low-density neutrophils (LDNs), a unique subpopulation of neutrophils, are garnering more attention due to their abundance is associated with the development and progression of numerous disease (Carmona-Rivera and kaplan M J. 2023; Sanchez-Pino et al. 2022; Torres et al. 2022). The discovery and identification of LDNs are based on the method of Ficoll-Hypaque density centrifugation. The Ficoll-Hypaque solution is with a density generally close to 1.077 g/ml (Basch et al. 1990; Abrams et al. 1985), which establishes a density gradient during centrifugation, allowing the settling of erythrocytes and neutrophils at the bottom of the tube, while mononuclear cells remain at the interface between the Ficoll-Hypaque and the plasma layer. However, in 1986, Hacbarth and Kajdacsy-Balla found a significant amount of neutrophils co-segregated with peripheral blood mononuclear cells (PBMCs) layer and described that as low buoyant density neutrophils (Hacbarth and Kajdacsy-Balla 1986). In 2003, these cells were found in donors treated with granulocyte-colony stimulating factor (G-CSF) using a similar identification method (Vasconcelos et al. 2003). Thus, LDNs are defined as granulocytes that appear in the PBMC layer after completing the density gradient centrifugation procedure. They can be isolated from monocytes using flow cytometry based on their CD15^+^, CD16^+^ and CD14^low^ expression (Denny et al. 1950).

Accumulating evidence shows that LDNs exhibit pathogenic characteristics across numerous diseases (Murad et al. 2022; Murru et al. 2023; Tay et al. 2020). For instance, higher levels of LDNs are associated with increased disease severity across a range of autoimmune diseases, such as psoriasis(Skrzeczynska-Moncznik et al. 2020), systemic lupus erythematosus (SLE)(Mistry et al. 2019), rheumatoid arthritis(Fresneda Alarcon et al. 2021), asthma(Fu et al. 2014), and anti-neutrophil cytoplasmic autoantibody (ANCA) vasculitis(Grayson et al. 2015). This association is mainly attributed to the pro-inflammatory properties of LDNs and their enhanced release of neutrophil extracellular traps (NETs). In cancer, the increased presence of LDNs, which inhibit T-cell function, correlates with a worse prognosis for patients (Saraiva et al. 2021; Futoh et al. 2023; Liu et al. 2017; Valadez-Cosmes et al. 2021). This includes an enhanced risk of tumor metastasis and recurrence, as well as reduced overall survival rates(Saraiva et al. 2021; Futoh et al. 2023; Liu et al. 2017; Valadez-Cosmes et al. 2021). Additionally, elevated numbers of LDNs linked to increased host tissue damage and worse clinical status in patients with infections and sepsis (Morrissey et al. 2021; Cabrera et al. 2021; Manna et al. 2019; Sun et al. 2022; Takizawa et al. 2021).

Beyond their increased presence in various pathological conditions, emerging evidence suggests that epigenetic modifications play a crucial role in regulating LDNs function (Coit et al. 2015). In SLE, for example, both normal-density neutrophils (NDNs) and LDNs exhibit significant DNA hypomethylation compared to healthy controls (Coit et al. 2015). While NDNs and LDNs from lupus patients show highly similar epigenetic profiles, a notable exception is the cytoskeleton-regulating gene *RAC1* (Donnelly et al. 2014), which shows hypomethylation in a CG site in lupus LDNs compared to autologous NDNs (Coit et al. 2015). Whether this RAC1 hypomethylation plays an important role in promoting the enhanced NETs formation observed in LDNs remains to be determined.

Overall, an excess of LDNs in pathological conditions may involve excessive inflammation, immune dysregulation, and impaired pathogen clearance. Evaluating LDNs levels may serve as a marker of dysregulated immune responses and disease severity, and targeting LDNs levels or functions may be a future therapeutic approach.

In this review, we address the origin, development, and heterogeneity of LDNs, and the roles of LDNs in system homeostasis and diseases. We will focus on the findings of the interaction between LDNs and other immune cells. We will then discuss potential novel therapeutic strategies of intervention in diseases by targeting LDNs.

## LDN biology

### Origins, development, and heterogeneity

In healthy individuals, LDNs make up around 5% or less of cells separated from the PBMC (Blanco-Camarillo et al. 2021; Hardisty et al. 2021). However, LDNs levels are elevated in conditions where there is a disruption of physiological homeostasis, including autoimmune disorders, cancers, severe infections, and trauma (Grayson et al. 2015; Morrissey et al. 2021; Goretti Riça et al. 2023; Cohen et al. 2019). Recent evidence has also implicated LDNs in neuroinflammatory conditions. Studies have shown significantly elevated LDNs levels in multiple sclerosis and neuromyelitis optica spectrum disorder (NMOSD) patients compared to healthy donors. Similarly, in neuropsychiatric SLE, LDNs may contribute to neuroinflammation through multiple mechanisms, including the production of proinflammatory cytokines and the release of factors such as matrix metalloproteinase- 9 (MMP- 9) and neutrophil gelatinase-associated lipocalin (NGAL) that can disrupt the blood–brain barrier (Sim et al. 2022).

Pinpointing the precise origin of LDNs poses a challenge due to the absence of surface markers that are completely different from NDNs. Currently, the more convincing argument is that the increased levels of LDNs mainly originate from two sources: partially from the degranulation and/or NETs release (non-lytic) of NDNs (Sun et al. 2022; Cho et al. 2023), and partially from the premature release of neutrophil progenitor and/or immature neutrophils from the bone marrow (Goretti Riça et al. 2023; Drifte et al. 2013; Montaldo et al. 2022). However, which of these two sources predominates depends on the specific pathological condition. Furthermore, the heterogeneity of LDNs is likely related to their different origins. LDNs derived from NDNs are primarily characterized as mature and activated neutrophils, while those originating from emergency granulopoiesis in the bone marrow are predominantly immature cells. Most studies in this area have characterized mature and immature subsets from PBMCs, and extensive research has validated the coexistence of immature and mature LDNs in vivo (Blanco-Camarillo et al. 2021; Tay et al. 2024; Bashant et al. 2021). The proportions and functional characteristics of these two subsets vary according to physiological and pathological settings (Tay et al. 2020; Hassani et al. 2020).

### Measurement of LDNs

**Fig. 1:**
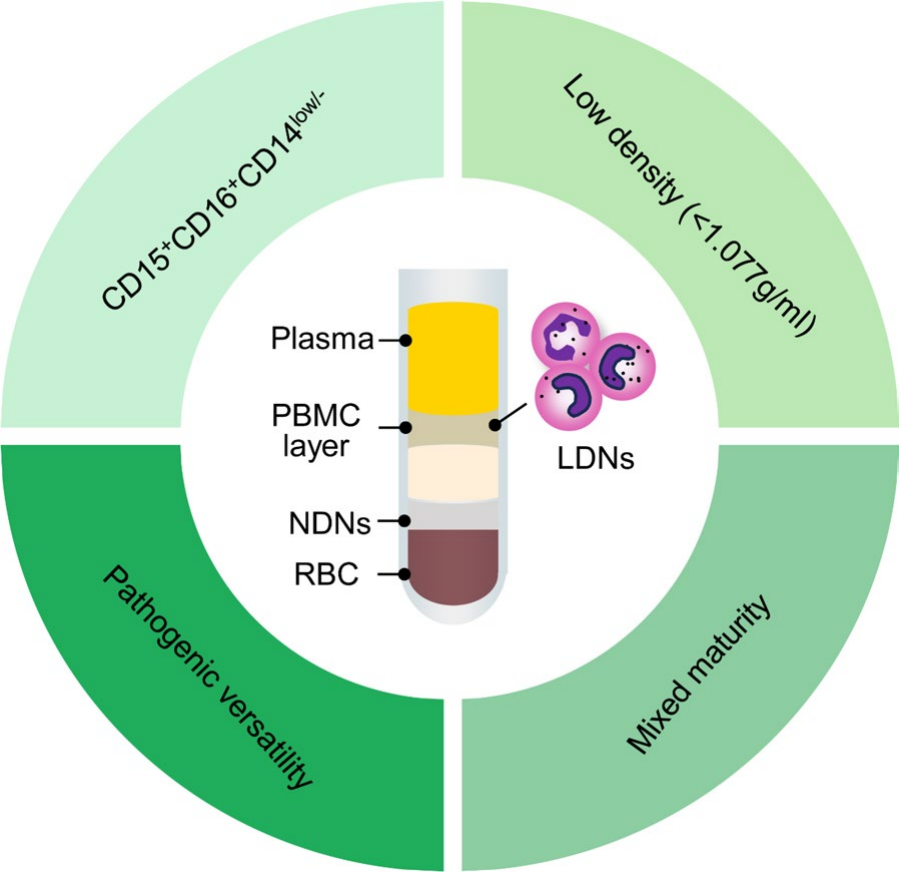
Key characteristics of LDNs. LDNs are found in the PBMC layer of a density gradient, which exhibit a low density (< 1.077 g/ml). LDNs are typically identified by their characteristic surface marker profile of CD15^+^, CD16^+^, and CD14^low/−^. Notably, LDNs display mixed maturity, indicating a heterogeneous population encompassing various developmental stages. Moreover, LDNs have been implicated in the pathogenesis of numerous diseases, highlighting their versatile role in various pathological conditions. *LDNs* low-density neutrophils, *PBMC* peripheral blood mononuclear cells, *NDNs* normal-density neutrophils, *RBC* red blood cell

Currently, one of the primary techniques relied upon for measuring LDNs is density gradient centrifugation. This method takes advantage of the lower buoyant density of LDNs, allowing them to be separated from NDNs in peripheral blood based on their different densities. After completing the density gradient centrifugation procedure, LDNs are found in the lower density fraction, typically above the Ficoll-Hypaque or Percoll interface, between the plasma and PBMC layers (Fig. 1) (Carmona-Rivera and kaplan M J. 2023). The second step involves identifying them from the PBMC population. Initially, researchers counted LDNs on slides after Giemsa-Wright staining or hematoxylin and eosin (H&E) staining (Hacbarth and Kajdacsy-Balla 1986; Vasconcelos et al. 2003) (Table 1), but this was undeniably a time-consuming and labor-intensive process. Subsequently, LDNs can be identified and quantified within the PBMC fraction using flow cytometry, based on the unique surface markers expressed on these granulocytes (Denny et al. 1950; Vasconcelos et al. 2006). Flow cytometry provides a rapid and sensitive measurement method. Common markers used for LDNs identification include CD15, CD16, and CD66b. Furthermore, when combined with surface markers indicative of activation and maturation status, such as CD10, CD11b, CD33, CD35, and CD63, this approach enables further functional characterization of LDNs, offering valuable insights into their activation state and potential roles in disease pathogenesis (Hassani et al. 2020; Seman and Robinson 2021).

**Table 1:** The debut of terms concerning LDNs

Term	LDNs
Year	1986
Surface markers	N
Identified methods	Giemsa-Wright stain and microscopy
Separation reagent	Ficoll-Hypaque
Condition	RA, SLE, ARF
Source of cells	Peripheral blood
Species	Human
References	Hacbarth et al. (Hacbarth and Kajdacsy-Balla 1986)

*N* Not mentioned, *LDNs* low-density neutrophils, *RA* rheumatoid arthritis, *SLE* systemic lupus erythematosus, *ARF* acute rheumatic fever

## Interaction of LDNs with other immune cells in the mechanism of diseases

**Fig. 2:**
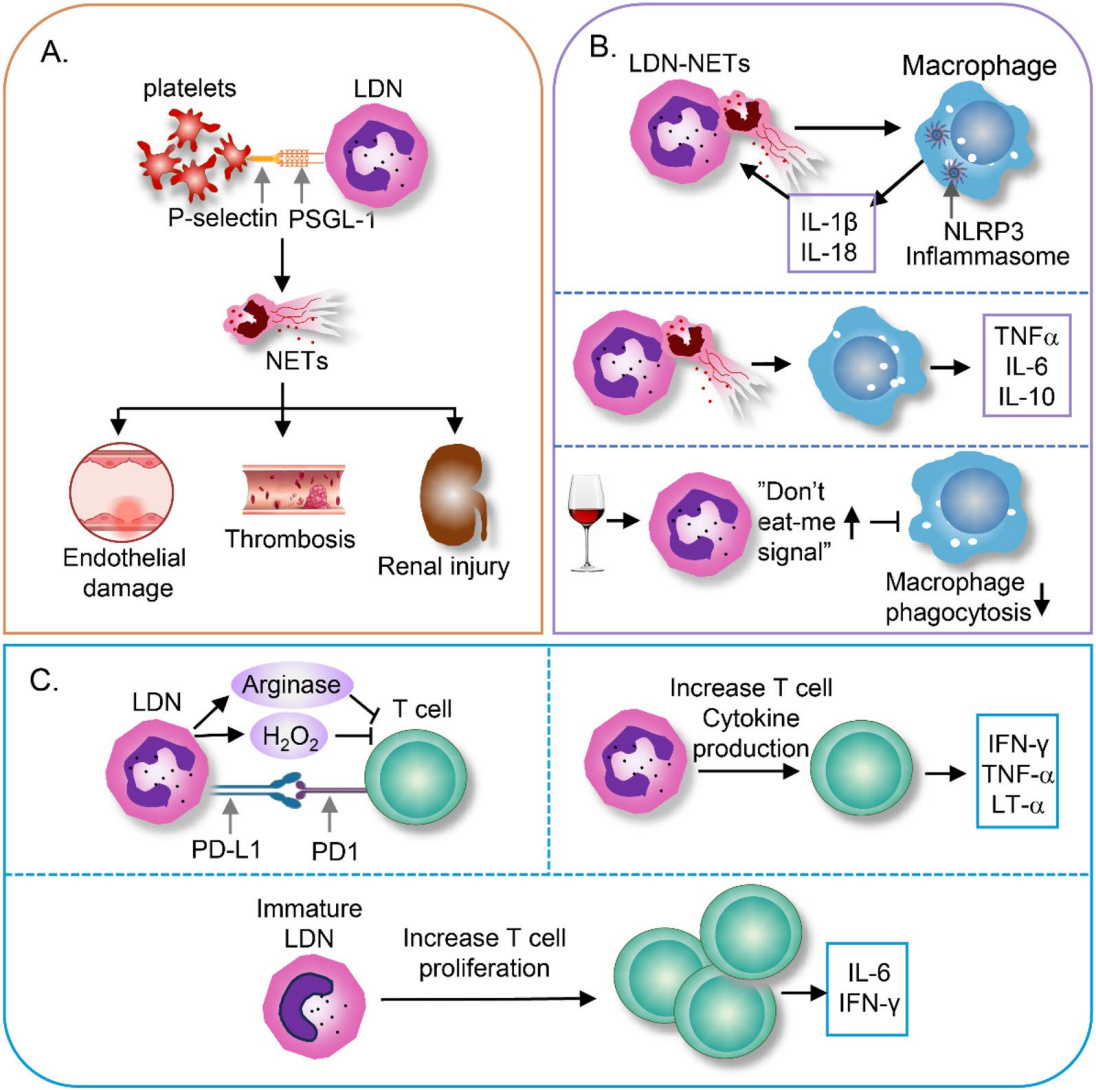
Interaction of LDNs with Other Immune Cells. **A**. LDN-platelet interaction. LDNs highly express PSGL- 1, which facilitates the formation of complexes between LDNs and platelets. LDN-platelet interactions significantly enhance the generation of NETs, which, in turn, leads to substantial tissue damage. **B**. LDN-macrophage interaction. LDNs in SLE patients show enhanced NET formation, activating NLRP3 inflammasomes in macrophages and increasing the production of pro-inflammatory mediators such as TNF-α, IL- 6, IL- 10, IL- 1β, and IL- 18, forming positive inflammatory feedback. On the other aspect, alcohol-induced LDNs exhibit elevated “don’t eat-me” signals, limiting macrophage phagocytosis of the LDNs. **C**. LDN-T cell interaction. LDNs exhibit complicated and context-dependent effects on T cell functions. LDNs can suppress T cell activity and proliferation through multiple mechanisms, including the production of H_2_O_2_, the release of arginine, and the expression of PD-L1. However, their impact varies significantly across different conditions. In SLE, activated LDNs induce a marked increase in the secretion of Th1 pro-inflammatory cytokines, notably IFN-γ, TNF-α, and LT-α. By contrast, in neonates, immature LDNs paradoxically enhance T cell proliferation and stimulate the production of cytokines such as IL- 6 and IFN-γ. *LDNs* low-density neutrophils, *LT-α* lymphotoxin alpha, *NDNs* normal-density neutrophils, *NETs* neutrophil extracellular traps, *PD-L1* programmed death-ligand 1, *PSGL- 1* P-selectin glycoprotein ligand- 1, *SLE* systemic lupus erythematosus

LDNs play a pivotal role in shaping the immune system (Blanco-Camarillo et al. 2021). For instance, they exert proinflammatory effects on T cells and induce high levels of proinflammatory cytokine production during SLE (Rahman et al. 2019). Besides, platelets preferentially bind to LDNs compared to NDNs, which is associated with the formation of NETs and increased inflammation in SLE (Tay et al. 2024). These indicate that LDNs engage in active communication with other innate and adaptive immune cells, potentially playing a crucial role in the pathogenic mechanisms (Fig. 2).

### LDN-platelet interaction

Platelets are anucleate cells and the second most abundant cellular component of blood, which have critical roles in preventing bleeding (Brass et al. 2016). Due to their widespread expression of functional immune receptors, platelets are not only essential for hemostasis but also critical for rapidly recruiting leukocytes and modulating immune responses (Scherlinger et al. 2023). Despite the extensive literature that has elucidated the platelet-neutrophil crosstalk and their subsequent activation, knowledge regarding the interplay between LDNs and platelets is scarce (Zarbock et al. 2007; Pircher et al. 2019). Recent emerging evidence indicates increased platelet adhesion to the surface of LDNs compared to NDNs (Tay et al. 2024), highlighting the necessity to characterize intersecting roles between LDNs and platelets in order to facilitate further studies.

Three studies utilizing microarray and sequencing analyses have demonstrated increased expression of CD41, a platelet marker, in LDNs compared to NDNs (Villanueva et al. 2011; Condamine et al. 2016; Monaco et al. 2019). This finding was obtained through a meta-analysis conducted by Tay et al. (Tay et al. 2024). This observation suggests that platelets may preferentially interact with LDNs, or LDNs may internalize platelets, under conditions of SLE, cancer, and health (Tay et al. 2024). Furthermore, Teague et al. (Teague et al. 2019) provided corroborating evidence through RNA sequencing, revealing higher gene expression of P-selectin and P-selectin glycoprotein ligand- 1 (PSGL- 1) in LDNs relative to NDNs. P-selectin is a platelet-specific receptor that binds PSGL- 1, facilitating tight adhesion between platelets and neutrophils (Polanowska-Grabowska et al. 2010). Notably, this study found that LDN-platelet interactions correlated with the promotion of vascular injury and disease severity in psoriasis skin disease (Teague et al. 2019). This suggests that LDN-platelets interactions might provide a potential therapeutic target for psoriasis and could extend to other disorders. For example, Dean et al. (Dean et al. 2022) reported that SARS-CoV- 2 infection triggers an increase in LDNs, which subsequently enhances the number of platelets bound to them and NETs forming. These changes could increase the risk of tissue damage and thrombosis (Dean et al. 2022). In addition, Tay et al. (Tay et al. 2024) recently reported that the formation of LDN-platelet aggregates was increased in lupus patients and was associated with nephritis. In this study, the authors suggested that these platelet-bound LDNs act a pivotal part in inflammation and tissue injury, partly because of the enhancement of NETosis (Tay et al. 2024). NETosis is a formation process of NETs in which activated neutrophils to release nuclear DNA, fibers, histones, and varied granule proteins into the extracellular environment (Thiam et al. 2020).

It is important to note that platelet activation can lead to neutrophil degranulation and increased buoyancy, potentially raising the frequency of LDNs in the PBMC fraction (Tay et al. 2024). This suggests that neutrophil degranulation is a potential mechanism of the generation of LDNs from NDNs. There exists some evidence that NDNs may undergo degranulation and transform into LDNs under many pathological conditions, including sepsis and autoimmune disorders (Sun et al. 2022; Deng et al. 2016; Mckenna et al. 2009). Overall, it seems that LDN-platelet interaction can be linked to the progression of disease, and this appears to be the case in SLE and psoriasis. However, the mechanisms behind the binding of platelets to LDNs and how this contributes to disease progression remain to be fully addressed. The increased formation of NETs may be a contributing factor, but further research is necessary to fully comprehend the process.

### LDN-macrophage interaction

Although intercellular communication exists between neutrophils and macrophages, studies directly linking LDNs and macrophages are limited (Shrestha and Hong 2023). Since LDNs exhibit heightened NETs formation under various pathological conditions (Skrzeczynska-Moncznik et al. 2020; Mistry et al. 2018; Carmona-Rivera et al. 2019; Hoogen et al. 2020), understanding the interplay between NETs and macrophages could provide valuable insights into elucidating the relationship between LDNs and macrophages. For instance, LDNs, as a distinct pro-inflammatory subset in SLE patients, exhibit a significantly enhanced capacity to generate NETs (Kahlenberg et al. 1950). Subsequently, the LDN-derived NETs possess the capability to activate the NLRP3 inflammasome in lupus macrophages, potentially establishing a vicious cycle of inflammation (Kahlenberg et al. 1950).

Barrera-Vargas et al. (Barrera-Vargas et al. 2018) were the first to identify ubiquitinated proteins in NETs that could alter calcium flux in macrophages by activating CXC chemokine receptor type 4 (CXCR4). Extracellular ubiquitin is linked to anti-inflammatory and regulatory responses in several diseases (Majetschak 2011; Garcia-Covarrubias et al. 2008). Notably, their study demonstrated LDN NETs exhibited the lowest levels of ubiquitinated proteins, compared to lupus and healthy NDN NETs (Barrera-Vargas et al. 2018). Consequently, when evaluating the response of SLE macrophages, the lowest increase in calcium flux was observed for LDN NETs (Barrera-Vargas et al. 2018). Moreover, macrophages perform a crucial function in eliminating NETs and apoptotic neutrophils (Schulz et al. 2021). However, NETs internalization by lupus macrophages is accompanied by an increased synthesis of pro-inflammatory mediators, including TNF-α, IL- 6, and IL- 10 (Barrera-Vargas et al. 2018).

There was a significant increase in LDNs within the PBMCs of alcohol-associated hepatitis (AH) patients in contrast to healthy controls (Cho et al. 2023). The elevated LDNs might represent a unique characteristic of AH, as they were not observed in the circulation of patients with non-alcoholic steatohepatitis (Cho et al. 2023). The important role of alcohol in promoting LDNs production was further confirmed in the alcohol-fed mice model (Cho et al. 2023). Instead of immature neutrophils generated by emergency granulopoiesis, alcohol-related LDNs are mature neutrophils, a fact corroborated by hyper-segmented nuclei and heightened expression of CD66b and CD11b (Cho et al. 2023). Of note, LDNs in AH patients display defective functions and diminished neutrophil homing/clearance. Specifically, LDNs exhibited decreased resting ROS levels, diminished phagocytic capacity, pronounced spontaneous NET formation, significantly reduced expression of “eat-me” signals, and were less phagocytosed by macrophages (Cho et al. 2023). Together, the further identification of the unique characteristics of LDNs can contribute to insight into the mechanisms for the increased inflammation and increased susceptibility to infection in patients with AH. These findings demonstrate that LDNs can exhibit distinct functional phenotypes in different pathological conditions, which may contribute to disease-specific immune responses.

### LDN-T lymphocytes interaction

T lymphocytes (T cells) are the primary cellular components of adaptive immunity. Their role is to orchestrate cell-mediated immune responses to maintain host health and prevent various diseases (Kumar et al. 2018). Typically, upon responding to various types of antigenic stimuli, T cells need to modulate their activation, clonal proliferation, and differentiation in a delicate balance (Chen 2023). The interaction between neutrophils and T cells has been extensively studied and is now described as a mainstream mechanism of inborn and adaptive immune crosstalk (Costa et al. 2019; Fan et al. 2023). However, the impact of LDNs on T cells remains a matter of debate, despite there are quite a few researchers who have observed that LDNs inhibit the proliferation and responsiveness of T cells (Manna et al. 2019; Hassani et al. 2020; Kumagai et al. 2020; Vlkova et al. 2019). Even some scholars consider LDNs and myeloid-derived suppressor cells (MDSCs) are the same type of granulocytes, as they both exhibit immunosuppressive functions in trauma, cancer, and other inflammatory diseases (Bowers et al. 2014; Schmielau and Finn 2001; Herteman et al. 2017). In fact, their developmental origins, tissue distribution, and characteristics differ significantly. MDSCs represent immature myeloid cells that arise from a partial block in normal myeloid differentiation in the bone marrow under pathological conditions (Gabrilovich and Nagaraj 2009). These cells can be detected in bone marrow, spleen, and peripheral blood (Schmielau and Finn 2001; Gabrilovich and Nagaraj 2009). In contrast, LDNs are primarily studied in the PBMC fraction and may develop from mature neutrophils (Sagiv et al. 2015). Notably, a small portion of MDSCs (1–5%) retain the ability to form myeloid-cell colonies and can differentiate into mature macrophages and dendritic cells under appropriate conditions (Bronte et al. 2000; Kusmartsev and Gabrilovich 2003; Li et al. 2004). While MDSCs are consistently immunosuppressive, LDNs represent a more heterogeneous population that can display immunosuppressive, proinflammatory, or immature phenotypes depending on the disease context (Ning et al. 2022). Given these distinct characteristics, it is appropriate to discuss them separately in immune modulation and disease pathogenesis. Here, we focus on the impact of LDNs on T cells (Table 2), as delving into these aspects of MDSCs is beyond the scope of this review.

**Table 2:** The impact of LDNs on T cells

Condition	Identifying markers	Status	Functional characteristics	Underlying mechanisms	References
Suppressor					
G-CSF–treated donors	N	N	Inhibited IFN-γ production by T cells	Associated with H_2_O_2_ production	Vasconcelos et al. (Vasconcelos et al. 2003)
G-CSF–treated donors	Gr1^+^, CD11b^−^, CD14^−^, SCA1^−/+^	Mature-type (more than 90%)	Inhibited IFN-γ production by T cells	Associated with H_2_O_2_ production	Vasconcelos et al.(Vasconcelos et al. 2006)
Tuberculosis	CD15 +	N	Inhibited T cell proliferation; Reduced IFN-γ production but did not reach statistical significance	Not attributable to IL- 10	La Manna et al. (Manna et al. 2019)
CVID	CD33^+^, CD14^−^, CD15^+^	N	Negative correlation between the frequency of LDNs and IFN-γ production by CD8^+^ T cells	N	Vlkova et al. (Vlkova et al. 2019)
Healthy	N	Increased immature cells than NDNs	Better in T-cell suppression than NDNs	N	Hassani et al. (Hassani et al. 2020)
Abdominal surgery	CD66b^+^	Mostly immature-type	Suppressed autologous T cell proliferation	May be related to PD-L1	Kumagai et al. (Kumagai et al. 2020)
Mild or asymptomatic infection with SARS–CoV–2	LIN^−^, HLA–DR^low/−^, CD11b +, CD14^−^, CD15^+^	N	Suppressed autologous T cell proliferation	May be related to GM–CSF and PD-L1	Siemińska et al. (Siemińska et al. 2021)
Tuberculosis	CD14^low^, CD15^+^	N	Inhibited IFN-γ production by T cells	May be related to PD-L1	Rao et al. (Rao et al. 2020)
Breast cancer	CD15 +	More activated phenotype	Reduced the activation and proliferation of effector T cells	May be related to PD-L1, ROS, and NETs	Saraiva et al. (Saraiva et al. 2021)
Healthy	CD66b^+^, CD16^+^	Heterogeneity	Suppressed CD4^+^ and CD8^+^ T cell proliferation similar to NDNs	N	Hardisty et al. (Hardisty et al. 2021)
NSCLC and ovarian cancer	CD45^+^, HLA-DR^−^, CD33^dim^, CD15^+^	Heterogeneity	CD45^high^ LDNs suppressed T cell proliferation, whereas CD45^low^ slightly increased that	N	Vanhaver et al. (Vanhaver et al. 2024)
Bystander					
Severe obesity	CD66^+^	N	Not suppress T cell proliferation	N	Sanchez-Pino et al. (Sanchez-Pino et al. 2022)
Activator					
Neonates	Lin^−^, CD66b^+^, CD64^+^, CD16^−^	Mainly immature-type	No antiproliferative effect on CD4 + T cells	N	Weinhage et al. (Weinhage et al. 1950)
SLE	CD45^+^, Lin^−^, HLA-DR^−^, CD11b^+^, CD33^+^, CD15^+^, CD14^−^	More activated phenotype	Not inhibit T cell proliferation, but induced proinflammatory cytokine production from CD4 + T cells	N	Rahman et al. (Rahman et al. 2019)
cGVHD	CD14^−^ CD16^+^	Heterogeneity	Enhance activated autologous T cell proliferative and cytokine responses	N	Matthews et al. (Matthews et al. 2021)

*N* Not mentioned, *H2O2* hydrogen peroxide, *CVID* common variable immunodeficiency disorders, *IFN-γ* interferon-γ, *PD-L1* programmed death ligand 1, *ROS* reactive oxygen species, *NETs* neutrophil extracellular traps, *NSCLC* non-small-cell lung cancer, *SLE* systemic lupus erythematosus, *cGVHD* chronic graft-versus-host disease

#### G-CSF-induced LDNs inhibit T-cell, which is related to the production of hydrogen peroxide (H_2_O_2_)

Extensive study has been conducted from neonates to adults to determine whether and to what extent LDNs exert immunosuppressive effects on T cells. While LDNs may show decreased ROS production in certain conditions like AH (Cho et al. 2023), their role in cancer-related immune responses presents different characteristics. In 2001, Schmielau and Finn (Schmielau and Finn 2001) observed that an abnormally large number of granulocytes copurify with PBMCs, during their investigation into the mechanism of T-cell function impairment in patients with advanced cancer. They demonstrated that the inhibition of T cell function in this group of granulocytes is primarily attributed to the production of H_2_O_2_ (Schmielau and Finn 2001). Although different experimental models were used, the results of Vasconcelos et al. (Vasconcelos et al. 2003) pointed in the same direction. They discovered that G-CSF was capable of transforming NDNs into LDNs, which are able to inhibit T-cell function through the production of H_2_O_2_ (Vasconcelos et al. 2003). Subsequently, Vasconcelos et al. (Vasconcelos et al. 2006) further found that LDNs produced by G-CSF treatment is able to inhibit 80% of IFN-γ production from T-cell in vivo and in vitro. These findings demonstrated that the immunosuppressive capacity of G-CSF was depended on LDNs, suggesting that the use of LDNs could be a potential therapeutic strategy for acute graft-versus-host disease (Vasconcelos et al. 2006).

#### Cancer-related LDNs suppress autologous T-cell proliferation

Since the discovery of the T-cell inhibitory effect of LDNs, researchers have been paying increasing attention to their role in regulating adaptive immunity in cancer. Multiple studies have consistently demonstrated that the number and proportion of LDNs significantly increase in cancer patients (Saraiva et al. 2021; Kumagai et al. 2020; Schmielau and Finn 2001). For example, Kumagai et al. (Kumagai et al. 2020) discovered a significant increase in LDNs following gastrointestinal cancer surgery, with these cells exhibiting distinct molecular and functional characteristics. Mechanistically, these post-operative LDNs display enhanced expression of PD-L1, L-selectin, and IL- 8 receptor, and demonstrate increased longevity compared to NDNs (Kumagai et al. 2020). The PD-L1 expression enables LDNs to suppress both autologous T-cell proliferation in response to TCR-CD3-mediated stimulation and the cytotoxicity of IL- 2-activated lymphocytes against tumor cells (Kumagai et al. 2020). Additionally, these LDNs demonstrate enhanced NET formation capacity, with these NETs efficiently trapping circulating tumor cells in vitro, suggesting a potential role in facilitating tumor cell survival and metastatic spread during the post-operative period (Kumagai et al. 2020).

While initial evidence for LDNs promoting tumor advancement and metastasis came from murine models (Hsu et al. 2019), recent studies in human cancer patients have provided more detailed mechanistic insights. Saraiva et al. (Saraiva et al. 2021) demonstrated that metastatic breast cancer patients exhibit a higher percentage of LDNs compared to non-metastatic patients, with estrogen receptor-positive (ER^+^) tumors showing particularly elevated LDNs levels. Their study revealed that these LDNs display enhanced expression of PD-L1 and increased levels of CD11b and CD66b compared to NDNs, indicating an activated immunosuppressive phenotype (Saraiva et al. 2021). Importantly, they uncovered a complex immunoregulatory network where LDNs demonstrate elevated production of TGF-β and CCL17, with the latter recruiting CCR4^+^ regulatory T cells (Tregs) (Saraiva et al. 2021). This creates a dual suppressive mechanism: direct inhibition of T cell activation and proliferation by LDNs (demonstrated through ex vivo co-culture assays), and indirect suppression via Treg recruitment (Saraiva et al. 2021). These findings, along with accumulating evidence from other studies (Futoh et al. 2023; Vanhaver et al. 2024), strongly suggest that elevated levels of LDNs correlate with unfavorable clinical outcomes regarding patient survival or remission.

LDNs might represent a heterogeneous cellular group comprising mature cells, immature cells, alongside activated/regulatory subpopulations, with their characteristics varying depending on specific pathological circumstances. This heterogeneity is particularly evident in cancer, where different studies have revealed distinct phenotypes and functions of LDNs (Saraiva et al. 2021; Kumagai et al. 2020; Schmielau and Finn 2001; Hsu et al. 2019). In breast cancer patients, CD11b and CD66b are more expressed in LDNs than in NDNs, indicating that LDNs are more activated than NDNs (Saraiva et al. 2021). Schmielau et al. (Schmielau and Finn 2001) demonstrated that such activated LDNs from patients with pancreatic, colorectal, and mammary carcinomas could suppress T cells through H_2_O_2_ release.

However, in post-operative gastrointestinal cancer patients (including esophageal, stomach, and colorectal cancers), Kumagai et al. (Kumagai et al. 2020) found that LDNs predominantly consisted of immature neutrophils, characterized by higher expression of CD62L and CXCR2 but lower levels of CD11b, CD16, and CD66b compared to NDNs. Similar findings were reported by Hsu et al. (Hsu et al. 2019) in a mouse model of liver metastases, where they observed increased mobilization of immature LDNs. Notably, these immature LDNs lacked the capacity to suppress T-cell proliferation in vitro (Hsu et al. 2019).

Recent work by Vanhaver et al. (Vanhaver et al. 2024) has helped reconcile these apparently conflicting observations. In lung cancer and ovarian carcinoma patients, they found that CD45^high^ LDNs (representing mature neutrophils) suppressed T-cell proliferation, whereas CD45^low^ LDNs (displaying immature morphology) were non-suppressive (Vanhaver et al. 2024).

Collectively, these findings suggest that cancer-related LDNs significantly impact tumor progression, with their immunosuppressive capacity likely dependent on their maturation state. However, additional investigations are required to confirm the T cell inhibitory effects of specific LDN subsets across different cancer types. If validated, this understanding could lead to novel targeted immunotherapies aimed at suppressing specific LDN populations or preventing their generation, thus potentially reducing their inhibitory impact on T cells.

#### Sepsis-related LDNs impaired T-cell function through L-arginine metabolism

Elucidating the mechanisms that lead to dysregulated host response to infection is a present challenge in sepsis investigation. The abnormal function of neutrophils in sepsis is a crucial contributor to the decline of the inherent immune system and the escalation of the disease (Ramoni et al. 2024; Zhu et al. 2022). The percentage of LDNs in PBMCs was measured at 37.3 ± 5.11% in sepsis patients, whereas the frequency in healthy individuals was 3.96 ± 0.44% (Sun et al. 2022). In sepsis, T-cell dysfunction through impaired delayed-type hypersensitivity and the reactivation of herpes simplex virus and cytomegalovirus in vivo was observed (Maclean et al. 1975; Kutza et al. 1998; Müller et al. 2006). Compared with sepsis patients without shock, septic shock patients exhibit significantly higher number of LDNs and more mature phenotypes (Darcy et al. 2014). These LDNs suppress T-cell function by mediating the CD3 zeta-chain downregulation. Moreover, the LDNs express arginase, which leads to the metabolism of L-arginine (Darcy et al. 2014). Study has shown that the addition of an arginase inhibitor can revive T-cell proliferation (Darcy et al. 2014). The exhaustion of arginine led to the obstruction of T cell signaling and suppression of function, shielding the fetus from immune rejection (Kropf et al. 2007). Overall, a comprehensive comprehension of the source and function of LDNs in sepsis could facilitate the diagnosis and treatment of sepsis in the future.

#### Tuberculosis-induced LDNs cause suppression of polyclonal T-cell proliferation

The inhibitory effect of LDNs on T cells has also been studied in the context of *M. tuberculosis* (TB) infection. Patients diagnosed with active TB harbored a notably higher number of LDNs in their peripheral blood when contrasted with healthy control individuals (Manna et al. 2019; Deng et al. 2016). Moreover, the percentage of LDNs escalated markedly even amidst an extension in the total neutrophils in the blood of active TB patients (Manna et al. 2019; Deng et al. 2016). In vitro, upon infecting PBMCs, which were harvested from TB patients, with Mycobacterium bovis Bacillus Calmette–Guérin, T-cell proliferation was assessed by the percentage of proliferating CD3^+^ T cells. LDNs markedly suppressed T-cell proliferation, whereas NDNs had no suppressive activities on T-cell response (Manna et al. 2019). Transcriptomic analysis demonstrated that IL- 10 was upregulated in LDNs. However, the addition of an anti-IL- 10 antibody did not alter the disparities in T-cell proliferation observed in the presence of LDNs or NDNs, suggesting that the impact of LDNs on T-cell proliferation in this study is independent of IL- 10(Manna et al. 2019). T-SPOT.TB (T-SPOT) assay represents the most widely utilized method worldwide to screen for or rule out TB infection (WHO 2018). Of note, LDNs can inhibit the production of IFN-γ in T cells via the highly expressed PD-L1, consequently diminishing the positivity rate of the T-SPOT assay (Rao et al. 2020). In contrast, the removal of LDNs from PBMCs significantly reduced the influence of the T-SPOT test (Zhang et al. 2017). The precise role of LDNs in TB pathogenesis, beyond T-cell suppression, still requires further elucidation.

#### COVID- 19-associated LDNs inhibit the antiviral defenses of T cells

The Coronavirus disease 2019 (COVID- 19) is an extremely transmissible respiratory illness, affliction stemming from severe acute respiratory syndrome coronavirus 2 (SARS-CoV- 2) infection (Xie et al. 2024). The prevalence of LDNs in the PBMCs of COVID- 19 patients was significantly increased compared to healthy controls (Cabrera et al. 2021). Strikingly, the median proportion of LDNs is 28.8% in early stage of COVID- 19 (within 24 h from hospital admission), whereas the median proportion for healthy controls is only 3.3% (Manunta et al. 2021). This proportion gap is further widened in critically ill COVID- 19 patients (Bruhn-Olszewska et al. 2022). Bruhn-Olszewska et al. (Bruhn-Olszewska et al. 2022) re-analyzed published single-cell RNA-seq data and added the status of loss of chromosome Y (LOY) to the annotated cells. Their findings indicated LDNs exhibit high levels of LOY and contribute to the development of COVID- 19 (Bruhn-Olszewska et al. 2022). In vitro, LDNs have been observed to inhibit the proliferation of autologous T cells and might result in lymphopenia and weakened immune response during COVID- 19 (Cabrera et al. 2021; Siemińska et al. 2021; Schulte-Schrepping et al. 2020). In addition, LDNs displayed a capability for the spontaneous generation of NETs, which primarily resulted in lung endothelial injury and vascular obstruction during SARS-CoV- 2 infection (Obermayer et al. 2021). Thus, the abundance of LDNs in the circulating blood could function as a supplementary clinical indicator to monitor COVID- 19 condition and progression.

#### Obesity-induced LDNs don’t substantially suppress T cell proliferation

Considering the crucial role of LDNs in various inflammatory conditions, Sanchez-Pino et al.(Sanchez-Pino et al. 2022) investigated the association between LDNs and chronic inflammation in individuals suffering from severe obesity. Their findings illustrated that individuals afflicted with morbid obesity exists an elevated number and frequency of LDNs (Sanchez-Pino et al. 2022). Following bariatric surgery, there was a reduction in the level of circulating LDNs, thereby facilitating the restoration of inflammatory homeostasis (Sanchez-Pino et al. 2022). Furthermore, having identified that the differential gene expression in LDNs between obese patients and normal controls were linked to immunosuppression via transcriptome analysis, they further evaluated the capacity of LDNs to suppress T cell proliferation through a co-culture assay. Surprisedly, LDNs did not substantially suppress T cell proliferation (Sanchez-Pino et al. 2022). These data suggested that obesity-induced LDNs exhibit pro-inflammatory capacities but did not manifest immunosuppressive functional characteristics. The potential plausible elucidation is that the activation of LDNs in obese individuals may be insufficient to completely induced the immunosuppressive functional properties.

#### LDNs activation properties

Weinhage et al. 1950 performed an investigation on heterogeneity and specific features of cord blood neutrophil in 136 neonates. Through microscopic and flow cytometric analyses, they identified LDNs as primarily immature cells with unique molecular signatures, particularly in their expression of S100 proteins. Notably, LDNs exhibited lower levels of S100 A12 compared to NDNs (Weinhage et al. 1950), with S100 A12 expression correlating with neonatal sepsis (Tosson et al. 2020). Functionally, neonatal LDNs demonstrated altered cellular activities, including reduced phagocytosis and delayed formation of NETs when compared to either adult or cord blood NDNs. These impairments in phagocytosis and NET formation are likely attributable, at least in part, to the immature state of LDNs (Weinhage et al. 1950). Interestingly, their interaction with T cells revealed a distinct immunomodulatory profile. Removal of LDNs from PBMCs did not enhance T-cell proliferation, conversely, the addition of LDNs at a 1:2 ratio actually promoted T-cell proliferation (Weinhage et al. 1950). This suggests that neonatal LDNs possess an activated immunophenotype, further highlighting the complexity of this cell subset.

Matthews et al. 2021 further studied and characterized immature LDNs in chronic graft-versus-host disease (cGVHD). The proportion of LDNs were substantially increased in cGVHD patients in comparison to healthy control subjects. Additionally, they discovered that LDNs were primarily constituted of CD10^−^ immature neutrophils and boosted the proliferative and cytokine responses of autologous T cells (Matthews et al. 2021).

The activated immunophenotype of LDNs has also been found in adult SLE patients (Rahman et al. 2019). Rahman et al*.* (Rahman et al. 2019) observed that LDNs did not inhibit CD4^+^ T cell proliferation and instead prompted proinflammatory cytokine profile in T cells. More precisely, in comparison to bead controls, SLE-LDNs were able to induce a significantly heightened secretion of the Th1 proinflammatory cytokines, such as IFN-γ, TNF-α, and lymphotoxin alpha (LT-α)(Rahman et al. 2019).

#### Comparison of LDN-T cells interaction and NDN-T cells interaction

While neutrophil-T cells interactions have been extensively documented (Shrestha and Hong 2023; Scapini and Cassatella 2014), a critical question remains unresolved: whether T cell-modulatory capacity is acquired by pre-existing neutrophils under specific conditions or executed by distinct neutrophil populations that emerge in pathophysiological environments and become specialized in either suppressing or promoting T cell responses(Moses and Brandau 2016; Scapini et al. 2016). The increased proportion of LDNs in various disease states offers potential insights into this question. LDNs were initially identified within the PBMC fraction of patients with various solid tumors or hematological malignancies (Saraiva et al. 2021; Kumagai et al. 2020; Schmielau and Finn 2001; Hsu et al. 2019; Vanhaver et al. 2024), and have subsequently been observed in numerous acute and chronic inflammatory conditions (Bowers et al. 2014; Darcy et al. 2014; Cloke et al. 2013; Pallett et al. 2015; Janols et al. 2014). Their effects on T cell function demonstrate remarkable complexity and context dependency. Current evidence indicates that LDNs can modulate T cell activity and proliferation through multiple mechanisms, including H_2_O_2_ production (Schmielau and Finn 2001), arginase release(Darcy et al. 2014), and PD-L1 expression (Kumagai et al. 2020). However, these effects exhibit significant variation across different pathological contexts. For instance, in SLE, activated LDN have been shown to induce a marked increase in Th1 pro-inflammatory cytokine secretion, particularly IFN-γ, TNF-α, and LT-α (Rahman et al. 2019). Conversely, in neonates, immature LDN paradoxically enhance T cell proliferation and stimulate the production of cytokines such as IL- 6 and IFN-γ (Weinhage et al. 1950).

Both LDN and NDN can interact with T cells through contact-dependent mechanisms, cytokine secretion, and NET formation under pathological conditions (Costa et al. 2019; Kalyan and Kabelitz 2014). However, their specific roles and mechanisms remain an active area of investigation. Understanding these interactions is crucial for elucidating the complex dynamics of neutrophil-mediated immune regulation.

### LDN-B lymphocytes interaction

LDNs have garnered attention for their role in type I interferons (IFN) production and their pro-inflammatory activities in SLE (Tay et al. 2020). The elevated quantities of B lymphocytes (B cells) exhibiting the DNA-binding protein ARID3a (A-T rich interacting domain 3a) correlates with IFNα manifestation and heightened disease severity in SLE (An et al. 2010; Popowski et al. 2014). The expression of ARID3a was first correlated with the generation of autoantibodies in B lymphocytes (Hayakawa et al. 2019). Furthermore, ARID3a is also expressed in LDNs, and studies have shown that its expression in these cells more strongly correlates with SLE disease activity indices (Ratliff et al. 2019). However, it remains uncertain whether ARID3a modulates IFN generation or whether IFN stimulates the expression of ARID3a in B cells and LDNs. The expression of ARID3a in LDNs exhibits a substantial correlation with SLE disease severity, suggesting its potential implication on disease pathogenesis. It is possible that ARID3a, being a vital transcription regulator, performs a pivotal function in SLE by regulating the synergy of LDNs and B cells. Further research may be necessary to fully understand whether LDNs directly interact with B lymphocytes and the exact mechanisms involved.

## Therapeutic potentials by targeting LDNs

While neutrophils play a crucial role in defending the body against pathogen-associated molecular patterns (PAMPs) or damage-associated molecular patterns (DAMPs), they may also contribute to severe inflammatory reaction and tissue injury (Chapple et al. 2000). LDNs have been found to exhibit a significant capacity for increased numbers and prominent pro-inflammatory properties in numerous inflammatory and autoimmune diseases (Ning et al. 2022; Ganesh and Joshi 2023). Thus, targeted therapy against pathogenic LDN subsets could potentially mitigate the tissue damage and inflammation caused while preserving the crucial roles of neutrophils in host defense and maintaining immune homeostasis. However, identifying specific markers for pathogenic LDNs, regulating LDNs generation, and reducing NETs formation remain challenges that need to be addressed. The heterogeneity of LDNs and the potential overlap in surface marker expression with other neutrophil subsets may complicate the development of selective therapies. Current research on targeted therapies for LDNs is primarily focused on inhibiting NETs formation (Carmona-Rivera and kaplan M J. 2023; Tay et al. 2020; Ning et al. 2022). NETs may trigger cytokine storms, leading to tissue damage; moreover, the myeloperoxidase (MPO) present in NETs can inhibit T cell activity, further promoting the immunosuppressive effects of LDNs in cancer (Németh et al. 2016; Odobasic et al. 2013). Therefore, targeting NET formation from LDNs holds significant therapeutic value.

Research in this field is making progress. Lood et al. (Lood et al. 2016) demonstrated that mitochondrial ROS stimulate the formation of LDN-derived NETs in both SLE and chronic granulomatous disease. The use of mitoTEMPO, a scavenger of mitochondrial ROS, decreased NETs formation and ameliorated kidney injury in mice prone to lupus (Lood et al. 2016). However, it's important to note that this drug is not specifically targeted to LDNs (Lood et al. 2016). Another investigation into SLE revealed that the voltage-dependent anion channel oligomerization inhibitor VBIT- 4 diminished mitochondrial RNA release, consequently inhibiting LDN NETosis in the same experimental model (Kim et al. 2019). These findings suggest that the exploration and development of drugs aiming at mitochondrial ROS of LDNs present promising therapeutic avenues. Tofacitinib, being the first-generation Janus kinase (JAK) inhibitor, has demonstrated notable efficacy in reducing NETs release and ameliorating lupus and associated vascular dysfunction in lupus-prone mice (Furumoto et al. 2017). Recently, a phase I clinical trial revealed that tofacitinib also can lower the levels of LDNs and circulating NETs in SLE patients (Hasni et al. 2021). This encouraging and inspiring advancement highlights the potential of therapeutically targeting LDNs. However, further studies are necessary to assess the effectiveness of this drug against additional pathogenic LDN subsets.

Recent studies have shown that elevated baseline LDNs are significantly associated with resistance to head and neck squamous cell carcinoma (HNSCC) and non-small cell lung cancer (NSCLC) immunotherapy, particularly in the context of immune checkpoint inhibitors (ICIs) (Arasanz et al. 2022; Arrazubi et al. 2023). In HNSCC patients undergoing immunotherapy, higher levels of LDNs were associated with significantly shorter progression-free survival (1.8 months vs. 10.9 months), indicating that LDNs may serve as a predictive biomarker for ICI response (Arrazubi et al. 2023). Similarly, in NSCLC, elevated baseline LDNs correlate with resistance to anti-PD- 1/PD-L1 monotherapy (Arasanz et al. 2022). Intriguingly, LDNs levels were not associated with resistance to anti-PD1 immunotherapy combined with chemotherapy, suggesting that this combination approach might deplete this cell population and permit anti-tumor responses (Arasanz et al. 2022). Of note, recent studies investigating JAK inhibition in combination with anti-PD- 1 therapy have demonstrated remarkable efficacy, achieving a 67% response rate in metastatic NSCLC patients (Mathew et al. 2024). Although this study did not specifically evaluate LDNs metrics, this combination therapeutic strategy provides a potential approach to overcome LDN-mediated immunotherapy resistance.

Despite these promising developments, several significant limitations exist in LDN-based therapeutic approaches. The heterogeneity of LDNs, which encompasses both immature and mature subpopulations with distinct phenotypic and functional characteristics, complicates efforts to achieve precise targeting. The current therapeutic strategies, such as targeting NET formation or mitochondrial ROS production, may affect both LDN and NDN populations, potentially compromising beneficial neutrophil functions. Furthermore, many of the molecular pathways and activation mechanisms observed in LDNs overlap with those in NDNs (Costa et al. 2019), complicating the development of effective therapeutic interventions.

## Conclusions and perspectives

The available data suggests that LDNs interact with immune cells via platelet binding, T cell modulation, and macrophage activation. The intricate and dynamic interplay between LDNs and other immune cells increasingly highlights their crucial role in immune regulation and provides insights into their contribution to the physiological functioning of the immune system and the pathogenesis of various disease conditions. Additionally, the critical role of LDNs in immune regulation highlights the importance of further research aimed at identifying the specific biomarkers and therapeutic targets associated with pathogenic LDNs across various diseases, elucidating the specific pathways and molecular mechanisms underlying their interactions with other immune cells.

As research in this field continues to evolve, the potential of LDNs in the realm of treatment of inflammation becomes increasingly evident. This review underscores the necessity for further investigation into the multifaceted roles of LDNs, aiming to harness their capabilities for more effective, targeted, and safe therapeutic interventions in inflammatory diseases. These insights will help develop targeted therapies that can reduce the detrimental effects of LDNs while preserving the beneficial roles of other neutrophil subsets in host defense and immune homeostasis.

## Data Availability

No datasets were generated or analysed during the current study.

## Abbreviations

AH: Alcohol-associated hepatitis
ANCA: Anti-neutrophil cytoplasmic autoantibody
ARID3a: A-T rich interacting domain 3a
cGVHD: Chronic graft-versus-host disease
COVID- 19: Coronavirus disease 2019
DAMPs: Damage-associated molecular patterns
G-CSF: Granulocyte-colony stimulating factor
H&E: Hematoxylin and eosin
H2O2: Hydrogen peroxide
HNSCC: Head and neck squamous cell carcinoma
IFN: Type I interferons
ICIs: Immune checkpoint inhibitors
JAK: Janus kinase
LDNs: Low-density neutrophils
LOY: Loss of chromosome Y
MDSCs: Myeloid-derived suppressor cells
MPO: Myeloperoxidase
MMP- 9: Matrix metalloproteinase- 9
NDNs: Normal-density neutrophils
NETs: Neutrophil extracellular traps
NGAL: Neutrophil gelatinase-associated lipocalin
NMOSD: Neuromyelitis optica spectrum disorder
NSCLC: Non-small cell lung cancer
PAMPs: Pathogen-associated molecular patterns
PBMCs: Peripheral blood mononuclear cells
PD-L1: Programmed death-ligand 1
PSGL- 1: P-selectin glycoprotein ligand- 1
SARS-CoV- 2: Severe acute respiratory syndrome coronavirus 2
SLE: Systemic lupus erythematosus
T-SPOT: T-SPOT.TB
TB: M. tuberculosis
